# SARS-CoV-2 infection and liver involvement

**DOI:** 10.1007/s12072-022-10364-1

**Published:** 2022-06-29

**Authors:** Mingjia Luo, Maria Pilar Ballester, Ugo Soffientini, Rajiv Jalan, Gautam Mehta

**Affiliations:** 1 Division of Medicine, University College London, London, UK; 2 Digestive Disease Department, Hospital Clínic, Universitario de Valencia, Valencia, Spain; 3 INCLIVA-Biomedical Research Institute, Valencia, Spain; 4 The Roger Williams Institute of Hepatology, Foundation for Liver Research, London, UK; 5 Liver Failure Group, UCL Medical School, Institute for Liver and Disease Health, University College London, Royal Free Campus, Rowland Hill Street, London, NW3 2PF UK

**Keywords:** SARS-CoV-2, COVID-19, Liver injury, Chronic liver disease, Cirrhosis, Alcohol-related liver disease, Autoimmune liver disease, Non-alcoholic fatty liver disease, Hepatitis B virus infection, Vaccination

## Abstract

The COVID-19 pandemic is the largest public health challenge in living memory. Patients with underlying liver disease have been disproportionately affected, experiencing high morbidity and mortality. In addition, elevated liver enzymes appear to be a risk factor for disease progression, even in the absence of underlying liver disease. Nevertheless, the mechanism of liver injury in SARS-CoV-2 infection remains largely unknown. This review aims to provide an overview of the mechanisms by which SARS-CoV-2 induces liver injury, and the impact of COVID-19 on cirrhosis, alcohol-related liver disease, autoimmune liver disease, non-alcoholic fatty liver disease, hepatitis B and C virus infection, liver-transplant recipients and patients with hepatocellular carcinoma. Finally, emerging data on vaccination in liver diseases is discussed, to help inform public health policy.

## Introduction

It is well known that coronavirus disease (COVID-19), caused by infection with severe acute respiratory syndrome coronavirus 2 (SARS-CoV-2), is an ongoing global pandemic [1]. The severe form of the illness is characterized by respiratory failure and hyperinflammation, with potential progression to multiorgan failure [1]. Advanced age, obesity and the presence of several comorbidities, such as cardiovascular disease (CVD), hypertension and diabetes have been linked with poor clinical outcomes in patients with COVID-19 infection [1].

Chronic liver disease (CLD), which is recognised as the 10th cause of death globally, contributes to a large health burden particularly among individuals of working age [1]. Patients with CLD often have several comorbidities such as obesity, type 2 diabetes mellitus (T2DM) and CVD, which overlap with COVID-19 risk factors, potentially predisposing to more severe disease through synergistic effects [2]. Given the dual burden of CLD and COVID-19 on health services, it is a priority for researchers to investigate the inter-relationship between SARS-CoV-2 infection and pre-existing liver disease and to make specific recommendations and guidance on therapy.

## Hepatotropism of SARS-CoV-2 virus

SARS-CoV-2 spike protein binds to Angiotensin-converting enzyme 2 (ACE2), which is a widely distributed, membrane-bound monocarboxypeptidase involved in processing of numerous peptides including angiotensin-II, but in the context of SARS-CoV-2 infection acts as a ‘receptor’ for SARS-CoV-2 to enter the host cell [2]. In healthy liver, the highest expression of ACE2 receptors appears to be in the biliary epithelium. Studies from liver-derived and induced pluripotent stem cell (iPSC)-derived organoids suggest that cholangiocytes are, in fact, highly susceptible to SARS-CoV-2 entry and replication [3, 4]. However, the pattern of liver abnormality noted with SARS-CoV-2 infection is not typical for cholestatic liver injury (see below), and direct evidence for cholangiocyte infection in COVID-19 is yet to be presented. It may be that low-level cholangiocyte infection does not lead directly to cholangiocyte cell death but is permissive for hepatocyte infection and may also cause delayed-onset biliary damage such as cholangiopathy which has been observed [5].

By contrast, hepatocytes appear to express low levels of ACE2 suggesting a potential low risk of SARS-CoV-2 entry, although hepatoma cell lines (e.g. Huh7, HepG2) and liver-derived organoids have been shown capable of supporting the complete life cycle of the virus [4, 6]. In vivo, electron microscopy supports the presence of intracellular virus particles within the hepatocyte, associated with mitochondrial swelling and structural damage, strongly suggesting direct cytopathy of SARS-CoV-2 in hepatocytes [7, 8]. This is particularly important since pre-existing liver disease is associated with *increased* expression of hepatocyte ACE2, thereby increasing the entry potential of SARS-CoV-2 into hepatocytes. For example, Meijnikman et al. describe increased hepatic ACE2 expression in patients with non-alcoholic fatty liver disease (NAFLD) [9], and Paizis et al. similarly find markedly increased hepatocyte ACE2 expression in hepatitis B virus (HBV)-related cirrhosis [10]. This increased expression of ACE2 is thought to be an injury response to liver fibrosis; indeed, ACE2 has been suggested as a therapeutic target for CLD [11]. Therefore, a potential mechanism for exaggerated SARS-CoV-2 liver injury in underlying CLD is due to greater burden of hepatocyte infection and consequently widespread hepatocyte cell death. An elegant demonstration of this is found in liver-derived organoids from cirrhotic NASH patients, which show markedly increased permissiveness to SARS-CoV-2 infection and pro-inflammatory gene expression compared to liver organoids from non-cirrhotic donors [12].

## SARS-Cov-2 related liver injury in the absence of chronic liver disease

The typical liver biochemistry of COVID-19 is of elevated transaminases rather than a cholestatic pattern, suggesting that cholangiocytes may potentially act as a viral reservoir without undergoing cell death. This is supported by post-mortem analysis of liver tissue from patients with SARS-CoV-2-related liver injury, which demonstrates predominant hepatocyte rather than cholangiocyte cell death [7]. The specific mechanisms underlying this difference in susceptibility are currently unexplained, but differential activity of inflammasome pathways in hepatocytes and cholangiocytes is one possible explanation.

**Fig. 1:**
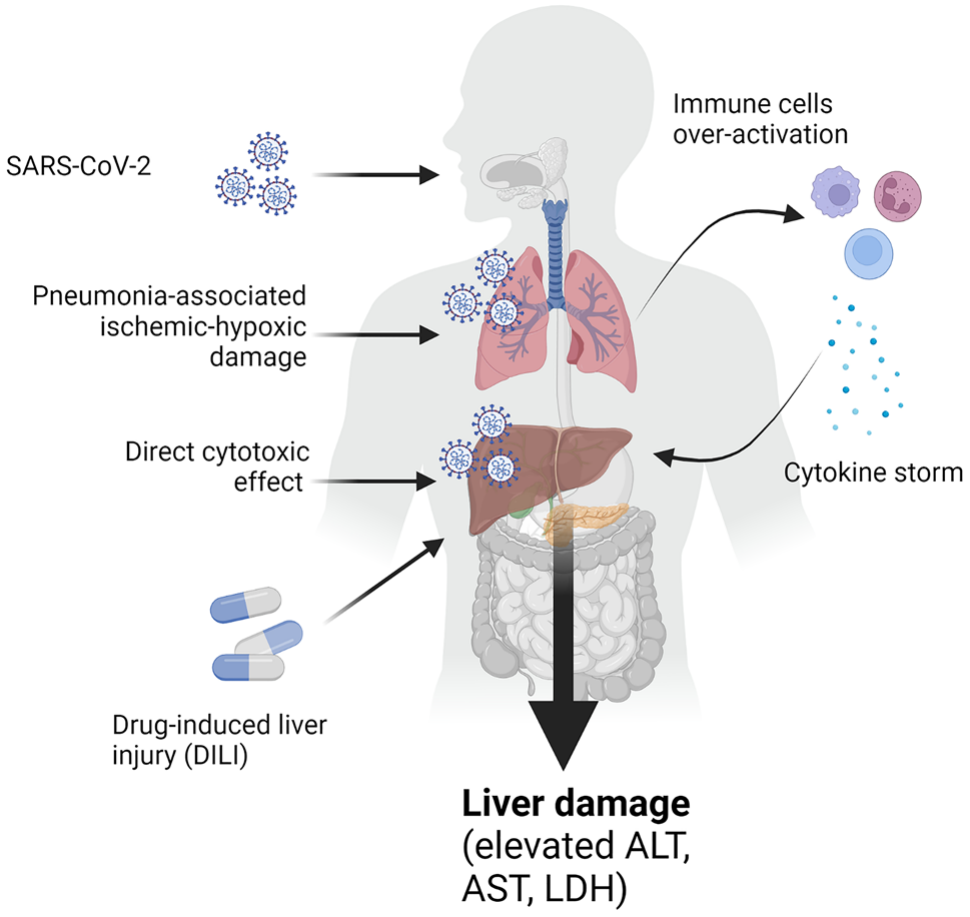
Covid-19 is associated with increased liver damage. SARS-CoV-2 infection in the lungs triggers ischemic/hypoxic damage as a result of pneumonia. In some patients, the antiviral response is disproportionate and results in over-activation of immune cells and consequent hyper-production of cytokines (cytokine storm). High levels of circulating cytokines, in particular IL-6, are associated with development of liver injury, which is exacerbated by direct cytotoxic effect of SARS-CoV-2 on the liver and by the hepatotoxicity associated with approved drugs for therapy against COVID-19

**Fig. 2:**
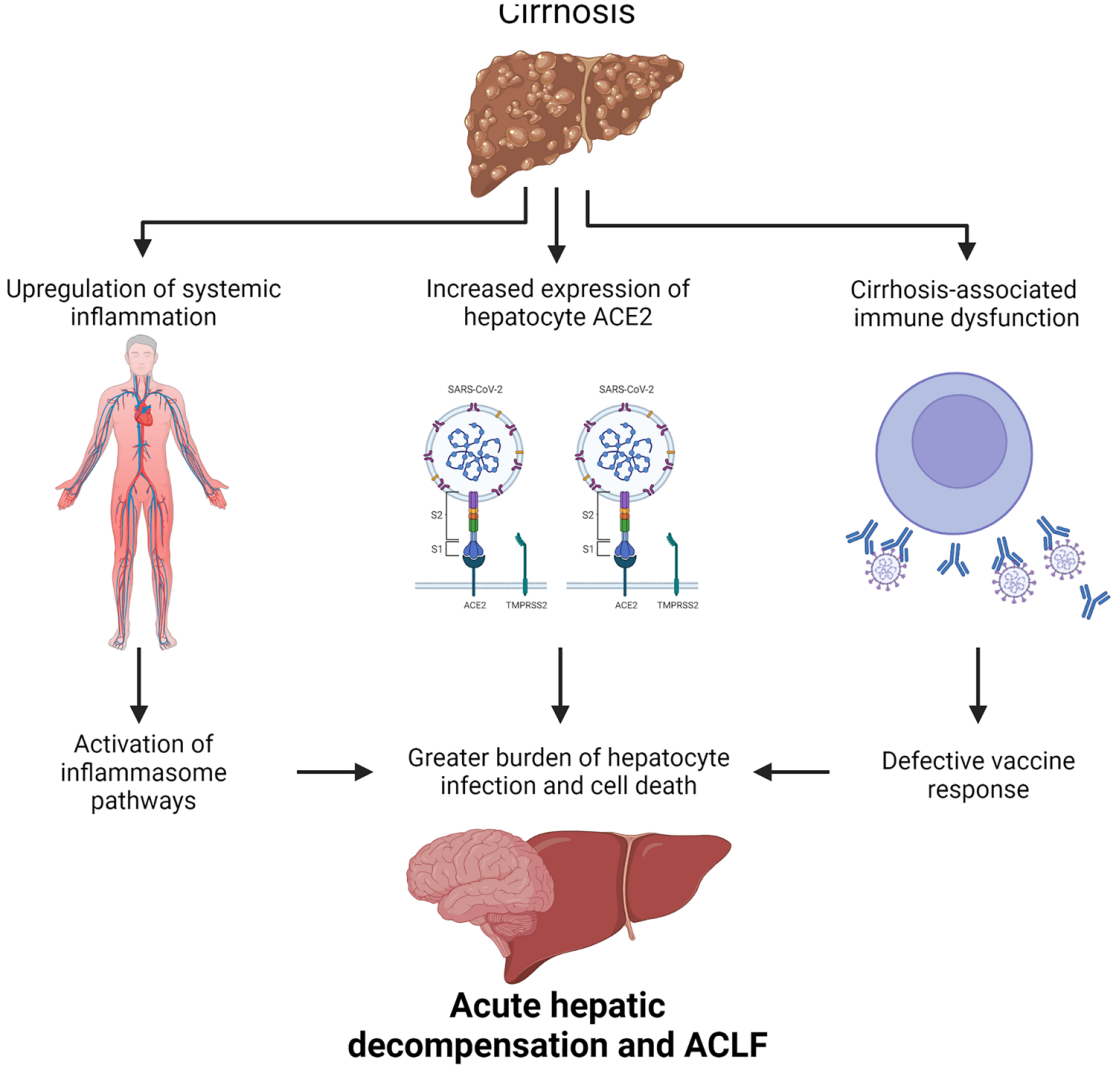
Patients with cirrhosis are at greater risk of developing acute hepatic decompensation upon SARS-CoV-2 infection, since they display weaker response to the vaccine resulting in lower levels of circulating antibodies against SARS-CoV-2 and also have increased expression of ACE2 on the hepatocyte membrane. ACE2 is not typically expressed on the surface of healthy hepatocytes but has elevated expression in cirrhosis permitting entry of SARS-CoV-2 into the cell. Cirrhosis is also associated with low-levels of circulating gut-derived bacterial products, which predispose to systemic inflammation and upregulation of inflammasome pathways. This results in sensitisation of hepatocytes to SARS-CoV-2 infection and subsequent pro-inflammatory cell death and immune responses

Several studies have reported the prevalence of elevated alanine aminotransferase (ALT) in COVID-19 to be 32–40% [13]. An association with systemic inflammation has been reported; Da et al. reported that the hyperinflammatory response in patients with severe COVID-19 was more pronounced among those with associated liver injury, and IL-6 was significantly elevated in the group with associated liver injury [14]. Further, McConnell et al. demonstrated elevated IL-6 in COVID-19 may lead to hepatic endotheliopathy due to sinusoidal endothelial cell dysfunction, contributing to liver injury [15]. However, data in this area are contradictory; the largest systematic review to date (23 studies) did not demonstrate a significant difference in liver enzymes or coagulation parameters in COVID-19 [16]. Therefore, as suggested above, liver injury in SARS-CoV-2 infection may be a reflection of a severe form of the disease with systemic inflammation or, in some cases, a degree of underlying CLD (Figs. 1, 2).

Drug-induced liver injury is a further potential mechanism for transaminase elevation in SARS-CoV-2 infection, either as a consequence of drugs used commonly in critical care or anti-viral drugs. An example of the latter is administration of lopinavir-ritonavir which, even in low-dose boost regimens, has been associated with the development of hepatotoxicity and should be used with caution in CLD. Both Remdesivir and Tocilizumab have also been reported to contribute to raised liver enzyme levels in 10% of cases. Most clinical trials of SARS-CoV-2 directed therapies excluded CLD patients, and thus clinical experience in CLD is limited to case reports.

To address the question of whether liver injury in COVID-19 is directly virus-related or a consequence of severe illness/systemic inflammation, a comparison with other severe respiratory viruses is useful. The pathobiology of influenza is well characterised; the virus is thought to only infect respiratory epithelial cells, and seasonal influenza does not typically cause liver injury. The more severe influenza A/H1N1 virus, which caused the 2009 pandemic, was associated with liver injury that correlated with degree of hypoxia and systemic inflammation [17]. More recently, Shafran et al. performed a retrospective comparison of ~ 1700 hospitalised patients with either influenza or COVID-19 at a single centre [18]. This group found similar predictors of disease severity on multivariate analysis in both groups, including liver transaminases, age, sex and degree of systemic inflammation. Therefore, these data support disease severity, either through hypoxia or inflammation/immune-mediated mechanisms, as being the major driver of liver injury in these respiratory infections. However, as discussed below, the same may not be true if underlying CLD is present. Indeed, case series’ support influenza A as being a cause for acute decompensation in patients with underlying cirrhosis [19, 20].

## SARS-CoV-2 infection on the background of chronic liver disease

### Cirrhosis and acute-on-chronic liver failure

Several studies have examined the clinical outcomes of SARS-CoV-2 infection on the background of cirrhosis. The characteristics of these studies are illustrated in Table 1.

**Table 1:** Summary of studies addressing Covid-19 outcomes in chronic liver disease and cirrhosis

Study	Study design	Location of study	Number of patients included	Main result
Marjot et al. (2021)	Multinational cohort study	29 countries	COVID-19 with non-cirrhosis CLD (*n* = 359); COVD-19 with cirrhosis (*n* = 386)	COVID-19 patients with cirrhosis were associated with higher rates for admission to ICU (*p* < 0.001), requirement to ICU (*p* < 0.001); renal replacement therapy (*p* = 0.002) and death (*p* = < 0.001) Overall mortality: COVID-19 with cirrhosis (32%) vs. COVID-19 with non-cirrhosis CLD (8%; *P* < 0.001)
Singh et al. (2020)	Multicentre research network study	United States	COVID-19 with non-cirrhosis CLD (*n* = 250); COVID-19 with cirrhosis (*n* = 50); COVID-19 without CLD (*n* = 2530)	Higher relative risk of mortality was found in COVID-19 patient with cirrhosis compared to COVID-19 patients without CLD (RR 4.6; 95% CI 2.6–8.3; *p* < 0.001)
Iavarone et al. (2020)	Multicentre retrospective cohort study	Italy	COVID-19 with without cirrhosis (*n* = 399); COVID-19 with cirrhosis (*n* = 50)	30-day mortality was significantly lower in COVID-19 patients without cirrhosis compared to those patients with cirrhosis (18% vs. 34%; *p* = 0.035) Decompensated cirrhosis was independently related to adverse outcomes of COVID-19
Ioannou et al. (2020)	Population-based study	North America	COVID-19 without cirrhosis (*n* = 9,826); COVID-19 with cirrhosis (*n* = 305)	COVID-19 patients with cirrhosis had significantly higher risk of hospitalisation (aHR1.37; 95% CI 1.12–1.66), death (aHR 1.65; 95% CI 1.18–2.30) and mechanical ventilation (aHR1.61; 95%: 1.05–2.46)
Sarin et al. (2020)	Multinational registry study	13 countries in Asia	COVID-19 with non-cirrhosis CLD (*n* = 185); COVID-19 with cirrhosis (*n* = 43)	20% of cirrhosis patients had either acute hepatic decompensation or acute-on-chronic liver failure 43% of mortality was found in patients with decompensated cirrhosis
Ge et al. (2021)	National electronic health dataset-based study	N/A	COVID-19 without cirrhosis (*n* = 29,446); COVID-19 with cirrhosis (*n* = 8,941); non COVID-19 without cirrhosis (*n* = 128,864); non-COVID-19 with cirrhosis (*n* = 53,476)	The presence of cirrhosis among COVID-19 patients was associated with 3.31 times adjust hazard of death (aHR 3.31; 95% CI 2.91–3.77; *p* < 0.01) COVID-19 infection was associated with 2.38 times adjust hazard of 30 days mortality among cirrhotic parents (aHR 2.38; 95% CI 2.18–2.59; *p* < 0.01)
Bajaj et al. (2021)	Multicentre retrospective cohort study	North America	Cirrhosis alone (*n* = 127); COVID-19 without cirrhosis (*n* = 108); COVID-19 with cirrhosis (*n* = 37)	The presence of cirrhosis was associated with increased mortality compared to COVID-19 alone (30% vs.13%, *p* = 0.03) No difference was found in mortality between COVID-19 with cirrhosis and cirrhosis alone (30% vs. 20%, *p* = 0.11)
Middleton et al. (2021)	Systematic review and meta-analysis	N/A	COVID-19 without cirrhosis (*n* = 31,082); COVID-19 with cirrhosis (*n* = 1,603)	The presence of cirrhosis was associated with increased all causes mortality (aOR 1.81; 95% CI 1.36–2.42)
Mandour et al. (2020)	Single centre retrospective study	United Kingdom	COVID-19 with cirrhosis (*n* = 10); non-COVID-19 with cirrhosis (*n* = 85)	COVID-19 infection was associated with longer hospitalisation stays with 11.5 days among cirrhotic patients (*p* = 0.047)
Suresh et al. (2020)	Hospital-based retrospective study	United States	COVID-19 without CLD (*n* = 1869); COVID-19 with non-cirrhosis CLD (*n* = 66); COVID-19 with cirrhosis (*n* = 21)	The presence of cirrhosis was associated with higher mortality rate (RR 2.1; 95% CI 1.33–3.62; *p* = 0.0022) compared to non-cirrhosis but no difference found in 30-day re-admission, ICU admission rate and intubation rate
Shalimar et al. (2020)	Single-centre retrospective study	India	COVID-19 alone (*n* = 722); COVID-19 with cirrhosis (*n* = 26); COVID-19 with NAFLD (*n* = 1); COVID-19 with extrahepatic portal venous obstruction (EHPVO)	COVID-19 was associated with higher mortality in cirrhotic patients compared to historical controls (42.3% vs. 23.1%, *P* = 0.077) Mechanical ventilation was associated with higher mortality in cirrhotic patients
Qi et al. (2020)	Multi-centre retrospective study	China	COVID-19 with cirrhosis survivor (*n* = 16); COVID-19 with cirrhosis non-survivors (*n* = 5)	Non-survivors were associated with higher rate of ICU admission (80% vs. 6.3%, *p* < 0.004), higher rate of non-invasive ventilation (60% vs. 6.3%, *p* = 0.028), higher rate of invasive mechanical ventilation (60% vs. 0%, *p* < 0.008)
Moon et al. (2020)	Multi-centre retrospective study	21 countries from 4 continents	COVID-19 with non-cirrhosis CLD (*n* = 49); COVID-19 with cirrhosis (*n* = 103)	Mortality was associated with the presence of cirrhosis with CTP-B (OR 4.90; 95% CI 1.16–20.61; *p* = 0.030) or CTP-C (OR 28.07; 96% CI: 4.42–178.46; *p* < 0.001) in patients with COVID-19
Liu et al. (2020)	Hospital-based retrospective study	China	COVID-19 with cirrhosis and CSPH (*n* = 6); COVID-19 with cirrhosis and non-CSPH (*n* = 11)	The presence of CSPH was not associated with CTP class (*p* = 0.125) and aetiology (*p* = 0.361) of cirrhosis, incubation (*p* = 0.472) of COVID-19

*CLD* chronic liver disease, *ICU* intensive care unit, *CTP* Child-Turcotte-Pugh, *RR* relative risk, *CI* confidence interval, *OR* odds ratio, *CSPH* clinically significant portal hypertension

References: Singh S, Khan A. Clinical Characteristics and Outcomes of Coronavirus Disease 2019 Among Patients With Preexisting Liver Disease in the United States: A Multicenter Research Network Study. Gastroenterology. 2020;159(2):768–771.e3; Iavarone M, D'Ambrosio R, Soria A, et al. High rates of 30-day mortality in patients with cirrhosis and COVID-19. J Hepatol. 2020;73(5):1063–1071; Ioannou GN, Liang PS, Locke E, et al. Cirrhosis and Severe Acute Respiratory Syndrome Coronavirus 2 Infection in US Veterans: Risk of Infection, Hospitalization, Ventilation, and Mortality. Hepatology. 2021;74(1):322–335; Sarin SK, Choudhury A, Lau GK, et al. Pre-existing liver disease is associated with poor outcome in patients with SARS CoV2 infection; The APCOLIS Study (APASL COVID-19 Liver Injury Spectrum Study). Hepatol Int. 2020;14(5):690–700; Mandour MO et al. 0415—Characteristics of sars-cov2 and liver cirrhosis- a single-centre experience in the United Kingdom. Hepatology, 2020;72(1 SUPPL):261A-262A; Suresh S, Siddiqui MB, Abu Ghanimeh M et al. Clinical outcomes in hospitalized COVID-19 patients with chronic liver disease and cirrhosis. Hepatology. 2020; 72:263A-263A; Shalimar, Elhence A, Vaishnav M, et al. Poor outcomes in patients with cirrhosis and Corona Virus Disease-19. Indian J Gastroenterol. 2020;39(3):285–291; Qi X, Liu Y, Wang J, et al. Clinical course and risk factors for mortality of COVID-19 patients with pre-existing cirrhosis: a multicentre cohort study. Gut. 2021;70(2):433–436; Moon AM, Webb GJ, Aloman C, et al. High mortality rates for SARS-CoV-2 infection in patients with pre-existing chronic liver disease and cirrhosis: Preliminary results from an international registry. J Hepatol. 2020;73(3):705–708; Liu F, Long X, Ji G, et al. Clinically significant portal hypertension in cirrhosis patients with COVID-19: Clinical characteristics and outcomes. J Infect. 2020;81(2):e178-e180; Marjot T, Moon AM, Cook JA, et al. Outcomes following SARS-CoV-2 infection in patients with chronic liver disease: An international registry study. J Hepatol. 2021;74(3):567–577; Ge J, Pletcher MJ, Lai JC; N3C Consortium. Outcomes of SARS-CoV-2 Infection in Patients With Chronic Liver Disease and Cirrhosis: A National COVID Cohort Collaborative Study. Gastroenterology. 2021;161(5):1487–1501.e5; Bajaj JS, Garcia-Tsao G, Biggins SW, et al. Comparison of mortality risk in patients with cirrhosis and COVID-19 compared with patients with cirrhosis alone and COVID-19 alone: multicentre matched cohort. Gut. 2021;70(3):531–536; Middleton P, Hsu C, Lythgoe MP. Clinical outcomes in COVID-19 and cirrhosis: a systematic review and meta-analysis of observational studies. BMJ Open Gastroenterol. 2021;8(1):e000739

In a European registry study of 745 patients with COVID-19, Marjot et al. found that cirrhosis patients had a significantly higher mortality rate than patients without cirrhosis (32% vs. 8%, *p* < 0.001) [21]. Similarly, a United States (US) multicentre study of 2780 patients with COVID-19 demonstrated that CLD patients had a relatively higher mortality rate than controls [relative risk (RR) 2.8, 95% confidence interval (CI) 1.9–4.0, *p* < 0.001], which was further increased in cirrhotic patients (RR 4.6, 95% CI 2.6–8.3; *p* < 0.001) [22]. A further retrospective study in 88,747 veterans, also in the US, demonstrated COVID-19 was associated with a twofold increased risk of mechanical ventilation, hospitalisation and mortality in cirrhotic patients compared to non-cirrhotic veterans [23]. In addition, mortality rate appears to be positively correlated with Child-Turcotte-Pugh (CTP) class [A (19%), B (35%) and C (51%)] [21], which is consistent with the 23.9%, 43.3% and 63.0% mortality rate reported by Mohammed et al. in CTP-A, CTP-B and CTP-C, respectively [24].

**Fig. 3:**
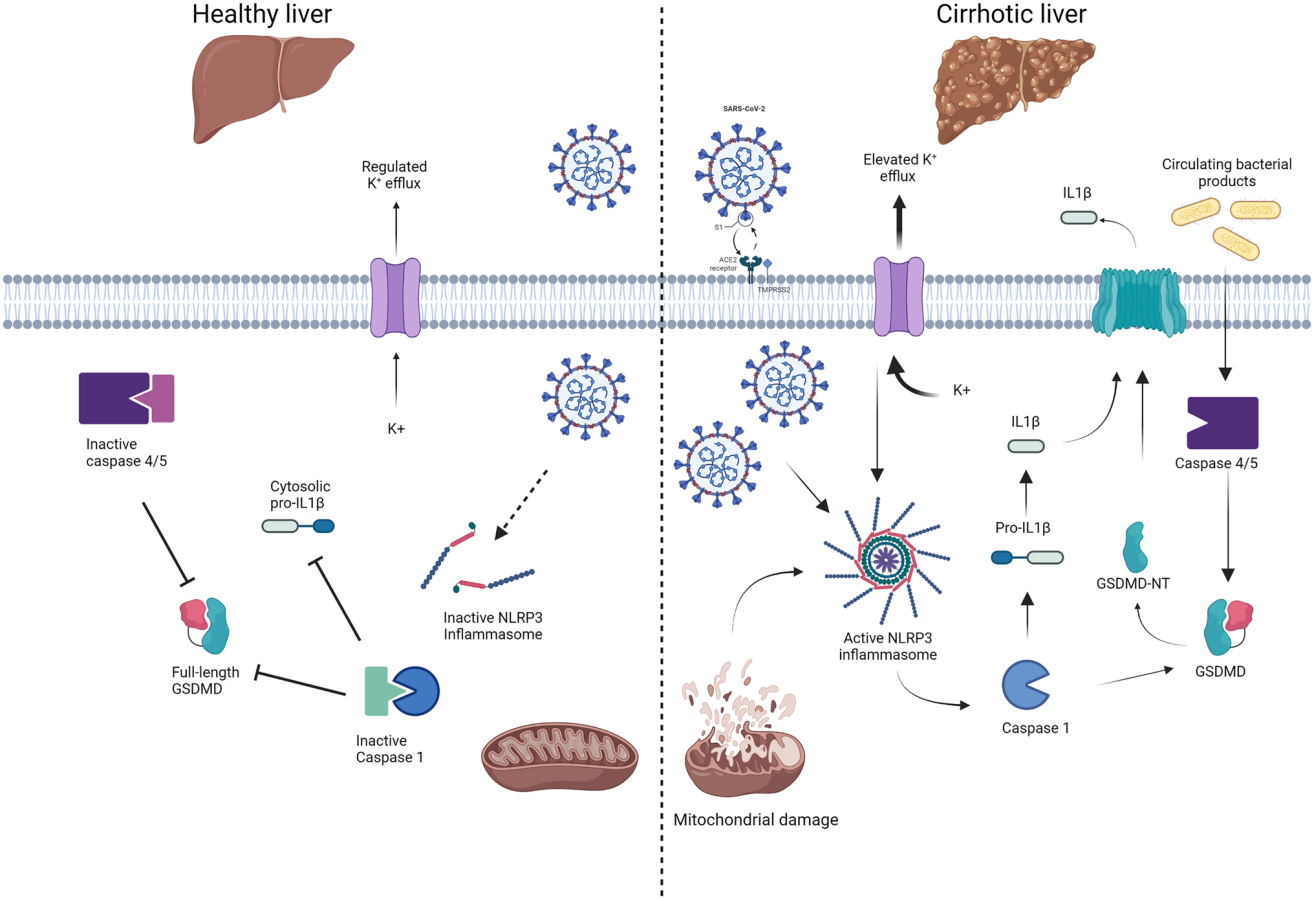
Upregulation of hepatocyte inflammasome signalling in cirrhosis predisposes to exacerbated cell death following SARS-CoV-2 infection. In cirrhosis (right panel) bacterial products (such as lipopolysaccharide) bind and activate Caspases-4/5 leading to cleavage of the dimeric protein Gasdermin-D (GSDMD). GSDMD N-terminal migrates to the plasma membrane, forming pores that allow the unregulated passage of damage-associated molecular patterns and electrolytes. K^+^ efflux and mitochondrial damage are also triggers of NLRP3 assembly, which in turns activates caspase-1 and leads to processing of pro-IL1β. Upon SARS-Cov-2 infection, viral proteins react with the already assembled NLRP3, leading to activation of downstream pathways. By contrast, in healthy liver (left panel), absence of ACE2 receptor delays the entry of SARS-CoV-2 into the cells. NLRP3 is present, but inactive, thus slowing the progress towards activation of pro-inflammatory caspases and processing of GSDMD and pro-IL1β

The mode of presentation of patients is also different in cirrhosis. Unlike the typical respiratory presentation in non-cirrhotic individuals, registry data from Asia supports a presentation with acute decompensation or acute-on-chronic liver failure (ACLF) in 20% of cirrhotic patients [25]. Data from Europe also support a presentation with acute decompensation in 46% of cirrhotic patients with COVID-19, with around half of these progressing to ACLF [21] (Fig. 2). Hepatocyte cell death is a key feature of the progression to acute decompensation or ACLF in cirrhosis [26]. From a mechanistic perspective, as shown in Fig. 3, the exaggerated release of inflammatory cytokines and the activation of inflammasome pathways in target cells are the likely triggers for cell death following SARS-CoV-2 infection [27]. In cirrhosis, the liver is further predisposed to injury, due to prior upregulation and activation of canonical and non-canonical inflammasome pathways [28, 29]. Specifically, the SARS-CoV-2 virus has been shown to interact and activate the canonical NLRP3 inflammasome and non-canonical pyroptosis inflammasome pathways, which are both drivers of cytokine storm [30, 31]. These inflammasome pathways have been shown to be upregulated in hepatocytes in cirrhosis, and hence the liver is ‘pre-sensitised’ to injury and cell death during SARS-CoV-2 infection in cirrhosis [29]. This subsequently leads to activation of pro-inflammatory caspases and the cell death executioner protein Gasdermin-D, which facilitates cytokine release and may also predispose to acute decompensation or ACLF (Fig. 3).

### Alcohol-related liver disease

Various studies have investigated the relationship between COVID-19 infection and alcohol-related liver disease (ARLD) (Table 2). Pre-existing ARLD has been linked to adverse outcomes in patients with COVID-19 infection (Table 2). Marjot and colleagues found that patients with ARLD had a higher risk of mortality from COVID-19 [odds ratio (OR) 3.11, 95% CI 2.12–4.55, *P* < 0.001] compared to other aetiologies [21]. This association remained significant after adjusting for several baseline characteristics including age, sex, disease severity and comorbidities (OR1.79, 95% CI 1.03–3.13, *p* = 0.040) [21]. Another multicentre observational cohort study of 867 patients with COVID-19 in the U.S. showed that ARLD was an independent predictor of overall mortality [hazard ratio (HR) 2.42; 95% CI 1.29–4.55; *p* = 0.006] [32].

**Table 2:** Summary of studies addressing Covid-19 outcomes in alcohol-related liver disease, autoimmune liver disease, non-alcoholic fatty liver disease, and hepatitis B infection

Study	Aim	Design	Location	Number of patients included	Main results
Alcohol-related liver disease					
Kim et al. (2021)	To identify the factors associated with adverse outcomes in patients with CLD who acquire COVID-19	Multicentre observational study	North America	COVID-19 with ALD (*n* = 94)	ALD independently predicted all-cause moryality (HR: 2.42; 95% CI 1.29–4.55; *p* = 0.006)
Marjot et al. (2021)	To determine the impact of COVID-19 on patients with pre-existing liver disease	Multinational cohort study	29 countries	Alive COVID-19 with ALD (*n* = 115); Dead COVID-19 with ALD (*n* = 64)	ALD was an independent risk factor for death from COVID-19 (adjusted OR 1.79; 95% CI1.03–3.13; *p* = 0.040)
Autoimmune liver disease					
Di Giorgio et al. (2020)	To explore the clinical features of SARS-CoV-2 infection in patients with AILD under immunosuppression	Phone-based survey	Italy	COVID-19 with immunosuppressed AILD (*n* = 4); immunosuppressed AILD only (*n* = 148)	Immunosuppression was not related to severe COVID-19 infection in patients with AILD
Efe et al. (2021)	To assess the clinical characteristics and outcomes of patients with AIH infected with COVID‐19	Multicentre cohort study	Europe and United States	COVID-19 with AIH (*n* = 110; 102 under immunosuppression)	Immunosuppression was not related to adverse outcomes of COVID-19 in patient with AIH AIH was not associated with higher hospitalisation (46.4% vs. 50.0%; *p* = 0.560), need for supplemental oxygen (38.2% vs. 42.2%; *p* = 0.553), all-cause mortality (10.0% vs. 11.5%; *p* = 0.852), or severe COVID-19 (15.5% vs. 20.2%; *p* = 0.231)
Non-alcoholic fatty liver disease					
Ji et al. (2020)	To examine the liver injury patterns and implication of NAFLD on clinical outcomes in Chinese patients with COVID-19	Hospital-based retrospective study	China	Stable COVID-19 (*n* = 163); stable COVID-19 with NAFLD (*n* = 42); progressive COVID-19 (*n* = 39); progressive COVID-19 with NAFLD (*n* = 34)	NAFLD was significantly associated with COVID-19 progression (OR 6.4; 95% CI 1.5–31.2). Patients with NAFLD presented higher risk of developing abnormal liver function from admission to discharge (11.1% vs. 70%; *p* < 0.0001) and longer viral shedding time (12.1 ± 4.4 days vs. 17.5 ± 5.2 days; *p* < 0.0001)
Zheng et al. (2020)	To investigate the association between MAFLD and COVID-19 severity	Multicentre retrospective cohort study	China	Obese COVID-19 with MAFLD (*n* = 45); non-obese COVID-19 with MAFLD (*n* = 21); Severe COVID-19 with obese and MAFLD (*n* = 17); severe COVID-19 with non-obese and MAFLD (*n* = 2)	Obese MAFLD patients had a sixfold increased risk of developing severe COVID-19 compared to non-obese MAFLD patients (adjusted OR 6.32; 95% CI 1.16–34.54; *P* = 0.033)
Targher et al. (2020)	To study whether MAFLD with increased non-invasive liver fibrosis scores are at higher risk of severe illness from COVID-19	Multicentre retrospective cohort study	China	COVID-19 with MAFLD and low FIB-4 (*n* = 44); with intermediate FIB-4 (*n* = 36); with high FIB-4 (*n* = 14)	Severe COVID-19 was associated with presence of intermediate (OR 4.32; 95% CI 1.94–9.59) or high FIB-4 scores (OR 5.73; 95% CI 1.84–17.9) among patients with MAFLD
Hepatitis B virus					
Liu et al. (2020)	To investigate liver function changes of COVID-19 patients with HBV infection, and how SARS-CoV-2 infection affects the course of chronic HBV infection	Retrospective cohort study	China	COVID-19 without chronic HBV infection (*n* = 51); COVID-19 with chronic HBV infection (*n* = 20)	Severe COVID-19 was similar in patients with and without HBV infection (30% vs. 31.4%; *p* = 0.97). Patient with HBV infection did not show longer median time to SARS-CoV-2 clearance compared with patients without HBV (21 days vs. 14 days; *p* = 0.1)
Chen et al. (2020)	To investigate the clinical characterizes of patients coinfected with SARS-CoV-2 and HBV	Hospital-based retrospective study	China	COVID-19 without HBV infection (*n* = 108); COVID-19 with HBV infection (*n* = 15)	HBV infection was associated with higher mortality rate compared to patients without HBV infection (13.3% vs. 2.8%)

*ARLD* alcohol-related liver disease, *AILD* autoimmune liver disease, *AIH* autoimmune hepatitis, *CLD* chronic liver disease, *NAFLD* non-alcoholic fatty liver disease, *MAFLD* metabolic associated fatty liver disease, *HBV* Hepatitis B virus, *OR* odds ratio, *CI* confidence interval, *HR* hazard ratio, *FIB-4* fibrosis-4

References: Kim D, Adeniji N, Latt N, et al. Predictors of Outcomes of COVID-19 in Patients With Chronic Liver Disease: US Multi-center Study. Clin Gastroenterol Hepatol. 2021;19(7):1469–1479.e19; Di Giorgio A, Nicastro E, Speziani C, et al. Health status of patients with autoimmune liver disease during SARS-CoV-2 outbreak in northern Italy. J Hepatol. 2020;73(3):702–705; Efe C, Dhanasekaran R, Lammert C, et al. Outcome of COVID-19 in Patients With Autoimmune Hepatitis: An International Multicenter Study. Hepatology. 2021;73(6):2099–2109; Ji D, Qin E, Xu J, et al. Non-alcoholic fatty liver diseases in patients with COVID-19: A retrospective study. J Hepatol. 2020;73(2):451–453; Zheng KI, Gao F, Wang XB, et al. Letter to the EditOR Obesity as a risk factor for greater severity of COVID-19 in patients with metabolic associated fatty liver disease. Metabolism. 2020;108:154244; Targher G, Mantovani A, Byrne CD, et al. Risk of severe illness from COVID-19 in patients with metabolic dysfunction-associated fatty liver disease and increased fibrosis scores. Gut. 2020;69(8):1545–1547; Liu J, Wang T, Cai Q, et al. Longitudinal changes of liver function and hepatitis B reactivation in COVID-19 patients with pre-existing chronic hepatitis B virus infection. Hepatol Res. 2020;50(11):1211–1221; Chen X, Jiang Q, Ma Z, et al. Clinical Characteristics of Hospitalized Patients with SARS-CoV-2 and Hepatitis B Virus Co-infection. Virol Sin. 2020;35(6):842–845

Several reasons can contribute to this increased risk of mortality from COVID-19 in patients with ARLD. Comorbidities such as malnutrition and metabolic syndrome are frequently present in patients with ARLD and have been linked with poor clinical outcomes in patients with COVID-19 infection [6]. Importantly, alcohol affects many organs, including the immune system, enhancing a proinflammatory response and the susceptibility to viral and bacterial infections [33].

Whether corticosteroid therapy administered for severe alcoholic hepatitis may aggravate severity of COVID-19 is controversial. A systematic review and meta-analysis evaluating the influence of corticosteroids on patients with coronavirus infection showed that length of hospitalization was longer (weighted mean difference = 6.31, 95% CI 5.26–7.37, *p* < 0.001) and adverse reactions such as bacterial infection (RR 2.08, 95% CI 1.54–2.81, *p* < 0.001) and hypokalemia (RR 2.21, 95% CI 1.07–4.55, *p* = 0.032) were more likely in the corticosteroid group. Nevertheless, the mortality of neither SARS-CoV (RR 2.56, 95% CI 0.99–6.63, *p* = 0.053, *I*^2^ = 77.4%, *p* < 0.001) nor MERS-CoV (RR 2.06, 95% CI 0.66–6.44, *p* = 0.213, *I*^2^ = 89.4%, *p* = 0.002) was correlated with corticosteroid therapy [34]. In addition, patients with severe conditions were more likely to require corticosteroids (RR 1.56, 95% CI 1.28–1.90, *p* < 0.001); result that was reproduced in the subgroup analysis of patients with SARS-CoV-2 infection in the meta-analysis (RR 2.36, 95% CI 1.31–4.28, *p* = 0.004, *I*^2^ = 29.1%, *p* = 0.235) [34] and in studies of SARS-CoV-2 infection (9.4% vs 1.2% in severe and non-severe COVID-19 *p* < 0.001) [33]. Therefore, the risk–benefit of corticosteroid administration should be assessed based on the severity of both alcoholic hepatitis and COVID-19 infection.

### Autoimmune liver disease

The effects of autoimmune liver disease (AILD) on COVID-19 remain unclear (Table 2). Corticosteroid therapy alone or combined with azathioprine is first-line treatment for AILD, both of which may predispose to viral infection. However, several studies reported a similar prevalence of COVID-19 infection in patients with autoimmune hepatitis (AIH) under immunosuppression than in the general population [35, 36]. In addition, several studies investigating the impact of immunosuppressive therapy on clinical outcomes of COVID-19 in patients with pre-existing AILD have not shown worse outcomes in severe SARS-CoV-2 infection (Table 2). In an American and European study of 34 centres, maintenance of immunosuppression during COVID-19 was not associated with poor prognosis [37]. Specifically, compared to patients without autoimmune hepatitis (AIH), patients with AIH did not show higher hospitalisation rates (46.4% vs. 50.0%; *p* = 0.560), need for supplemental oxygen (38.2% vs. 42.2%; *p* = 0.553) or all-cause mortality (10.0% vs. 11.5%; *p* = 0.852) [37]. Similarly, in a telephone-based survey conducted in Italy, Di Giorgio et al. demonstrated that the use of immunosuppressive therapy in AILD patients was not associated with more severe COVID-19 compared to the general population [35].

### Non-alcoholic fatty liver disease

Given the relationship between metabolic syndrome and severity of SARS-CoV-2 infection, several studies have examined the effect of non-alcoholic fatty liver disease (NAFLD) on COVID-19 prognosis (Table 2). In a retrospective Chinese study of 202 consecutive patients with COVID-19, Ji et al. demonstrated that NAFLD is associated with higher risk of progression to severe COVID-19 (OR6.4, 95% CI 1.5–31.2), higher likelihood of abnormal liver function from admission to discharge [70% (53/76) vs*.* 11.1% (14/126), *p* < 0.001] and longer viral shedding time (17.5 ± 5.2 days vs*.* 12.1 ± 4.4 days, *p* < 0.001) compared to patients without NAFLD [38]. In another Chinese study including 66 consecutive patients with NALFD, obese patients presented a sixfold higher risk of severe COVID-19 infection (adjusted OR 6.32; 95% CI 1.16–34.54; *p* = 0.033) after controlling for sex, smoking, age, dyslipidemia, hypertension and diabetes [39]. In addition, Targher et al. demonstrated that intermediate fibrosis-4 (FIB-4) scores (OR4.32, 95% CI 1.94–9.59) or high FIB-4 scores (OR5.73, 95% CI 1.84–17.9) correlate with higher severity of COVID-19 [40].

### Hepatitis B virus infection

Several studies have investigated the impact of chronic hepatitis B virus (HBV) infection in COVID-19 patients with controversial results (Table 2). In a Chinese retrospective study of 347 patients, neither the probability of severe COVID-19 (30% vs. 31.4%, *p* = 0.97) nor the time to clearance of SARS-CoV-2 (21 days vs. 14 days, *p* = 0.1) differed between patients with and without HBV [41]. On the contrary, a second retrospective study carried out by Chen and colleagues found that patients with HBV infection had significantly higher mortality compared with patients without HBV infection (13.3% vs. 2.8%) [42]. Hence, larger prospective studies, accounting for severity of underlying liver disease, are required to determine the impact of HBV infection on COVID-19 outcomes.

Specific concerns have been raised regarding HBV reactivation in COVID-19 patients receiving immunosuppressive treatment. Liu et al. demonstrated that, regardless of corticosteroid use, patients with COVID-19 and chronic HBV coinfection (HBsAg + ve and/or HBV DNA + ve) had an elevated risk of HBV reactivation [41]. Regarding resolved HBV infection, Rodriguez-Tajes et al. conducted a prospective study of 490 patients in Spain, finding that the risk of HBV reactivation in patients with resolved HBV (majority HBsAg −ve/anti-HBc + ve) was low despite use of immunomodulatory therapy for COVID-19 [43]. Not all patients received nucleoside analogues for HBV prophylaxis, and therefore, specific recommendations on the use of HBV prophylaxis cannot be inferred but given the safety of these drugs prophylaxis is considered a prudent option.

Various studies have also investigated the relationship between anti-viral treatment for HBV and outcomes from SARS-CoV-2 infection. In a Korean retrospective study of 19,160 patients with COVID-19, Choe et al. demonstrated that anti-viral treatment among HBV-infected patients was not associated with reduced mortality, hospitalisation stay, or rate of ICU admission compared to patients without antiviral therapy [44]. Of note, the antiviral-treated group showed higher proportion with progression to liver failure than their counterparts (4.3% vs. 1.3%; *p* = 0.032). However, this group also had a higher proportion of cirrhotic patients (46.4% vs. 12.1%, *p* < 0.001), which is a major confounder. Indeed, Kang et al. also found no difference in risk of death or adverse outcome between groups with, or without, antiviral treatment [45]. Therefore, it remains unclear if there is an independent effect of HBV antivirals on SARS-CoV-2 progression.

### Hepatitis C virus infection

A number of studies have also examined the impact of hepatitis C virus (HCV) infection on patients with COVID-19. A large, US-based retrospective study of 1193 patients found that HCV infection was significantly associated with both higher rates of in-hospital mechanical ventilation [58% (29/50) vs. 39% (449/1143), *p* = 0.003] and increased all-cause mortality [56% (28/50) vs. 34% (386/1143), *p* = 0.003] compared to patients without HCV infection [46]. These groups had similar prevalence of cirrhosis (2.5% vs. 5%, *p* = 0.534), hence this relationship was considered independent of severity of liver disease. A similar Romanian study demonstrated that active HCV patients (HCV RNA viral load > 0.015 × 10^3^U/L) had higher rates of hospitalisation (61.2% vs. 40.0%, *p* < 0.0001), length of stay (28 days vs. 21 days, *p* = 0.0142), mechanical ventilation (26.3% vs. 13.1%, *p* = 0.0007) and all-cause mortality (48.3% vs. 11.5%, *p* < 0.0001) compared to non-active HCV patients, although cirrhosis was not accounted for as a covariate in this study [47]. A further US based study demonstrated contrary results, using HCV patients and propensity-matched HCV negative controls, finding no difference in ICU admission or mortality although an independent effect of liver fibrosis (FIB-4 score) on hospitalisation rate was found [48]. Therefore, currently it remains unclear if HCV has a specific disease-modifying effect on SARS-CoV-2 infection and further large studies are required.

In terms of the effect of HCV antivirals, a Spanish study examined the effect of anti-viral treatment for HCV on SARS-CoV-2 infection [49]. This group found that the SARS-CoV-2 infection rate was 293 cases/100,000 patients with HCV infection receiving DAA therapy, which was lower than the contemporaneous infection rate in Madrid and Catalonia (771 cases-1,045 cases/100,000 habitants), hence the researchers concluded that DAA therapy may have a partial anti-SARS-CoV-2 effect. However, this study is limited by selection bias and retrospective design, and consequently larger studies will be needed to investigate any association between DAAs and SARS-CoV-2 infection.

### Post-liver transplantation

The management of liver transplant (LT) patients during the COVID-19 pandemic has been particularly challenging due to the need for immunosuppressive therapies. Several studies have addressed the outcomes of COVID-19 in patients following liver transplantation (Table 2). In a prospective Spanish study including a consecutive cohort of 111 LT patients with COVID-19, Colmenero et al. found that the epidemiological curve and geographic distribution of infection overlapped widely between the LT and general populations [50]. Moreover, the overall mortality rate was 18%, which was lower than in the matched general population after adjusting for sex and age [50]. Similarly, data from two international registries (COVID-Hep and SECURE-Cirrhosis), including 151 LT recipients with SARS-CoV-2 infection, demonstrated that LT did not significantly increase the risk of death [absolute risk difference 1·4% (95% CI  − 7.7 to 10.4)]. In addition, immunosuppressive therapy was not a risk factor for mortality unlike age [OR1·06 (95% CI 1.01–1.11)] per 1 year increase), serum creatinine concentration [OR1·57 (95% CI 1.05–2.36) per 1 mg/dL increase] and non-liver cancer [OR18·30 (95% CI 1.96–170.75)], which were associated with death [51]. Similarly, an Italian study showed that long-term LT recipients were more prone to severe disease than short-term LT patients, suggesting that immunosuppression *per se* does not increase the risk of severe COVID-19. Instead, the presence of comorbidities typically observed in long-term recipients, such as metabolic complications, may be responsible for the increased risk of severe COVID-19 [52]. A similar study conducted by Webb and colleagues confirmed these findings by demonstrating that death was significantly associated with both age and Charlson Comorbidity Index, whereas no association was found with immunosuppressive therapy [53]. However, as in the general population, degree of liver injury remains an independent predictor of mortality. Rabiee et al. evaluated this in 112 LT patients, showing that after adjusting for sex, ethnicity, race, age, comorbidities, immunosuppression and time since transplantation, presence of liver injury was independently associated with both higher risk of ICU admission (OR 7.93, 95% CI 1.75–35.69, *p* = 0.007) and higher overall mortality (OR 6.91, 95% CI 1.68–28.48, *p* = 0.007) [54].

### Hepatocellular carcinoma

Several studies have also investigated the impact of hepatocellular carcinoma (HCC) in patients with COVID-19 (Table 3). In a study of 745 patients with COVID-19, Marjot et al. found that presence of HCC was not independently associated with mortality compared to patients without HCC (OR 1.46, 95% CI 0.67–3.18, *p* = 0.346) [21]. By contrast, a more recent study from Kim and colleagues demonstrated in a different cohort that HCC is an independent predictor of death in patients with COVID-19 (HR 3.31, 95% CI 1.53–7.16) [32]. Possible reasons for this difference relate to the size of the cohorts; the Kim et al. is larger than that from Marjot et al. (867 patients vs. 745 patients), although comprises fewer HCC cases than the Marjot study (2.5% vs. 6.4%). Additionally, the Kim et al. study was a multi-centre US-based cohort, whereas the Marjot study was an international registry with potentially greater heterogeneity.

**Table 3:** Summary of studies addressing Covid-19 outcomes in hepatocellular carcinoma

Study	Aim	Design	Location	Number of patients included	Main results
Hepatocellular carcinoma					
Marjot et al. (2021)	To determine the impact of COVID-19 on patients with pre-existing liver disease, which currently remains ill-defined	Multinational cohort study	29 countries	Alive COVID-19 with HCC (*n* = 34); died COVID-19 with HCC (*n* = 14)	Presence of HCC was not independently associated with mortality compared to patients without HCC (OR1.46; 95% CI 0.67–3.18; *p* = 0.346)
Kim et al. (2021)	To identify the factors associated with adverse outcomes in patients with CLD who acquire COVID-19	Multicentre observational cohort study	North America	Alive COVID-19 with HCC (*n* = 10); died COVID-19 with HCC (*n* = 9); Severe COVID-19 with HCC (*n* = 18); non-severe COVID-19 with HCC (*n* = 3)	HCC was one independent predictor of death in patients with COVID-19 (HR:3.31; 95% CI 1.53–7.16)

*HCC* hepatocellular carcinoma, *CLD* chronic liver disease

References: Marjot T, Moon AM, Cook JA, et al. Outcomes following SARS-CoV-2 infection in patients with chronic liver disease: An international registry study. *J Hepatol*. 2021;74(3):567–577; Kim D, Adeniji N, Latt N, et al. Predictors of Outcomes of COVID-19 in Patients With Chronic Liver Disease: US Multi-center Study. *Clin Gastroenterol Hepatol*. 2021;19(7):1469–1479.e19

### COVID-19 vaccination in chronic liver disease and liver transplant recipients

Important recent data on covid-19 vaccination suggest decreased efficacy and durability of protection in patients with cirrhosis. Specifically, retrospective observational data from the us veteran’s affairs database including 20,037 patients with cirrhosis who received at least 1 dose of covid-19 mrna vaccine (51% mrna-1273, 49% bnt162b2 mrna) demonstrated delayed and reduced protection from covid-19 infection in cirrhosis compared to previously reported data in healthy individuals (64.8% reduction after 1-dose, 78.7% reduction after 2 doses) [55]. Moreover, the degree of protection was further reduced in decompensated cirrhosis (50.3%) compared with compensated cirrhosis (66.8%) [55]. Registry data collected between March and august 2021 also demonstrate the potential for severe covid-19 in cirrhosis despite vaccination. Although only limited cases numbers are reported (21 infections in CLD patients after ≥ 1 vaccine dose), 33% of reported cases required hospitalisation [56].

Regarding serological responses to vaccination, there are relatively few prospectively collected data. In NAFLD, data from a Chinese cohort suggest that serological responses remain intact. This multicentre 381 NAFLD patients, without a history of SARS-CoV-2 infection, demonstrated that neutralizing antibodies against SARS-CoV-2 were detected in 95.5% of patients after 2 doses of vaccination [57]. However, most of the patients in this study did not have advanced fibrosis or cirrhosis. By contrast, emerging data from CLD patients suggest decreased immunity following vaccination. Data from a US cohort of 79 patients with cirrhosis (10 decompensated), demonstrated that cirrhotic patients have significantly lower anti-Spike serological responses (32% lower in all cirrhotic patients, and 54% in decompensated patients) compared to controls [58]. Similarly, an Italian cohort had significantly lower anti-Spike titres in cirrhosis patients than controls after both the first [13.9 (0.4–12,500) vs. 43.1 (0.4–345) U/mL, *p* = 0.001] and second doses of vaccine [1034 (0.4–12,500) vs 1520 (259–12,500)U/mL, *p* = 0.05]. Moreover, those with decompensated cirrhosis, HCC and undetectable anti-Spike Ab after first dose had significantly lower anti-Spike titres after the second dose of vaccine [637 (0.4–12,500) vs. 1377 (0.4–12,500) U/mL, *p* = 0.01; 1116 (0.4–7,500) vs. 1810 (0.4–12,500) U/mL, *p* = 0.02; 469 (0.4–4,780) vs. 3,908 (91.7–12,500) U/mL, *p* < 0.001, respectively]. The evaluation of spike-specific T cell responses after the first and second dose also indicated that patients with cirrhosis mount a weaker IFN-g and IL-2 response compared to healthy subjects [59].

For patients with LT, a similar rate of hospitalisation was seen in registry data, albeit also with low numbers of cases reported. Moon et al., report a series of 19 LT recipients (only 6 fully vaccinated) with 6 hospitalizations (32%), including 3 severe cases (16%) resulting in mechanical ventilation and 2 (11%) resulting in death [56]. Immunogenicity among LT recipients also seems to be reduced compared with controls (positive IgG Spike-protein in 47.5% post-LT vs. 100% controls, *p* < 0.001) with significantly lower antibody titre (95.41 vs. 200.5AU/ml, *p* < 0.001) 10–20 days after receiving the second BNT162b2 vaccine dose [60]. Other studies exploring the safety and immunogenicity of COVID-19 vaccination in LT patients are summarized in Table 4.

**Table 4:** Effect of COVID-19 vaccination in liver transplant recipients

Study	Aim	Study design	Vaccine brand	Number of patients	Main result
Boyarski et al. (Transplantation 2021)	To evaluate safety of the first dose	Participants completed a detailed online questionnaire 1 week following their first dose	Pfizer/BioNTech (50%) or Moderna (50%)	187 solid organ transplant recipients	No cases SARS-CoV-2, acute rejection, neurological diagnoses (Guillain–Barr syndrome, Bell’s Palsy, or neuropathy), or allergic reactions requiring epinephrine. Two cases of a new infection (acute-on-chronic pouchitis and influenza A) requiring treatment. Local site reactions included mild pain (61%), mild redness (7%), and mild swelling (16%). Systemic reactions such as fever and chills were uncommon (4% and 9%), although more-than-baseline fatigue was reported by 38%, headache by 32%, and myalgias by 15%
Boyarsky et al. (JAMA 2021)	To study the proportion of positive antibody response after a single dose	Antibodies to the S1 domain and anti-RBD of the SARS-CoV-2 spike protein at a median of 20 days after the first dose	mRNA (52% BNT162b2 vaccine and 48% mRNA-1273 vaccine)	436 solid organ transplant recipients	Antibody was detectable in 17% (95% CI 14–21%) of patients. Factors associated with lower response were anti–metabolite immunosuppression therapy (37% vs 63%; adjusted IRR 0.22; *P* < .001) and older age (adjusted IRR 0.83 per 10 years; *P* = .002). mRNA-1273 showed higher response than BNT162b2 (69%vs 31%; adjusted IRR 2.15; *p* = 0.003)
Timmermann et al. (Vaccines 2021)	To investigate the immune response alongside the influence of underlying diseases and immunosuppressive regimen	Anti-spike-protein-IgG testing at least 21 days after complete SARS-CoV-2 vaccination	BNT162b2 (*n* = 114), mRNA-1273 (*n* = 3) and JNJ-78436735 (*n* = 1)	118 liver transplant recipients	78% developed anti-spike-protein-IgG antibodies. Alcoholic liver disease before transplantation (*p* = 0.006) and mycophenolate mofetil-based regimen (*p* < 0.001) were associated with lower response rate. All patients weaned off immunosuppression were seropositive
Rashidi-Alavijeh et al. (Vaccines 2021)	Analyze immunogenicity	SARS-CoV-2 IgG against the Spike glycoprotein in a median of 15 days after receiving two doses of the vaccine	BNT162b2	43 liver transplant recipients and 20 healthcare workers as control group	79% liver transplant recipients developed antibodies (100% in the control group; *p* = 0.047). The median IgG titter was significantly lower in the liver transplant recipients (216 vs. > 2080 BAU/mL in controls, *p* = 0.0001). Mycophenolate mofetil was associated with a reduced response compared to the other liver transplant patients (45.5% vs. 90.6%, *p* = 0.004)
Rabinowich et al. (J Hepatol, 2021)	To asses vaccine immunogenicity and safety	SARS-CoV-2 IgG antibodies against the Spike-protein and Nucleocapsid-protein 10–20 days after receiving the second dose	BNT162b2	80 liver transplant recipients and 25 healthy controls	47.5% liver transplant patients presented positive serology (vs 100% in controls; p < 0.001). Antibody titer was also significantly lower in this group (mean 95.41 AU/ml vs. 200.5 AU/ml in controls, *p* < 0.001). Predictors for negative response were older age, lower eGFR, high dose steroids and mycophenolate mofetil. No serious adverse events were reported in either group
Thuluvath et al. (J Hepatol, 2021) (29)	To asses vaccine immunogenicity and safety	Antibody responses to spike protein, 4 weeks after complete vaccination	2 doses of mRNA vaccines or after the single dose of Johnson & Johnson	62 liver transplant recipients	Antibody levels were undetectable in 11 patients and suboptimal (median titter 17.6, range 0.47–212 U/ml) in 27 patients. Liver transplantation, use of 2 or more immunosuppressive therapies and vaccination with Johnson & Johnson were associated with poor response. No patient had any serious adverse events
Boyarsky B et al. (JAMA 2021)	To asses antibody response after the second dose	Anti-spike serologic testing which tests for the receptor-binding domain of the SARS-CoV-2 spike protein at a median of 29 days after dose 2	mRNA	658 solid organ transplant recipients	Antibody was detectable in 54%. Among the 473 receiving antimetabolites, 8% had response after dose 1 and dose 2; 57% had no response after dose 1 or dose 2; and 35% had no response after dose 1 but subsequent antibody after dose 2. Among the 185 participants not receiving antimetabolites, 32% had response after dose 1 and dose 2; 18% had no response after dose 1 or dose 2; and 50% had no response after dose 1 but subsequent antibody after dose 2
Boyarsky B et al. (Transplantation, 2021)	To quantify the antispike antibody response to the Janssen vaccine and compare it to recipients of the mRNA series	Antibodies anti-RBD of the spike protein at 1 month after COVID-19 vaccine	Janssen (*n* = 12) and mRNA group (*n* = 725)	12 solid organ transplant recipients	Anti-RBD antibody was detectable in only 17% of participants who received the Janssen (vs 59% in mRNA series, *P* = 0.005), with lower odds (aOR0.11; *P* = 0.006) of developing anti-RBD antibodies than those who completed the mRNA series. Median anti-RBD Ig titers in the Janssen group were significantly lower than the mRNA group (2.39 versus 106.9 U/mL; *P* = 0.047)
Herrera et al. (Am J Transplant 2021)	To study cellular and humoral immune response	IgM/IgG antibodies and ELISpot against the S protein 4 weeks after receiving the second dose	mRNA-1273	58 liver and 46 heart recipients	64% developed IgM/IgG antibodies and 79% S-ELISpot positivity. 90% developed either humoral or cellular response (87% in heart and 93% in liver recipients). Factors associated with vaccine unresponsiveness were hypogammaglobulinemia and vaccination during the first year after transplantation. Local and systemic side effects were mild or moderate, and none presented donor-specific antibodies or graft dysfunction after vaccination
Kamar et al. (NEJM 2021)	To report the humoral response	Antibodies to SARS-CoV-2 spike protein in patients who were given three doses	BNT162b2	101 solid organ transplant recipients (78 kidney, 12 liver, 8 lung or heart, and 3 pancreas)	The prevalence of anti–SARS-CoV-2 antibodies was 0% before the first dose, 4% before the second dose, 40% before the third dose, and 68% 4 weeks after the third dose. Among the 59 patients who had been seronegative before the third dose, 44% were seropositive at 4 weeks after the third dose. All 40 patients who had been seropositive before the third dose were still seropositive 4 weeks later; their antibody titers increased from 36 to 2676 1 month after the third dose (*P* < 0.001). Patients who did not have an antibody response were older, had a higher degree of immunosuppression, and had a lower eGFR. No serious adverse events were reported, and no acute rejection episodes occurred
Guarino et al. (Clin Gastroenterol Hepatol 2022)	To evaluate immunogenicity and to identify factors associated with negative response	Anti-Spike protein IgG-LIAISON SARS-CoV-2 S1/S2-IgG chemiluminescent assay at 1 and 3 months after 2-dose vaccination	BNT162b2	492 liver transplant recipients and 307 controls matched by age and sex	Detectable antibodies were observed in the 75% of patients with a median value of 73.9 AU/mL after 3 months from 2-dose vaccination. Older age (> 40 years, *p* = 0.016), shorter time from liver transplantation (< 5 years, *p* = 0.004), and immunosuppression with antimetabolites (*p* = 0.029) were associated with non-response. Liver transplant recipients showed antibody titers lower than the control group (103 vs 261 AU/ml, *p* < 0.0001). Both in controls and transplant patients there was a trend of correlation between age and antibody titers (correlation coefficient: − 0.2023 and − 0.2345, respectively)
Toniutto (J Hepatol 2022)	To assess the long-term antibody response in liver transplant compared to controls	Anti-RBD IgG and anti-nucleocapsid protein IgG measurements at the one, four and six months after the second dose	Pfizer-BioNTech BNT162b2 vaccine	143 liver transplant and 58 controls	Among COVID-19 naïve, 66.4%, 77%, and 78.8% were anti-RBD positives at one, four and six months following the second dose, while 100% of controls were positive at 4 months (*p* < 0.001). The median anti-RBD titter at four months was significantly lower (32U/ml) in COVID-19 naïve than in controls (852 U/ml, *p* < 0.0001). Mycophenolate (*p* < 0.001), ascites (*p* = 0.012), and lower leukocyte count (*p* = 0.016) were independent predictors of anti-RBD negativity at six months

*IRR* incidence rate ratio, *eGFR* estimated glomerular filtration rate, *anti-RBD* Anti-receptor binding domain protein

Therefore, well-conducted prospective studies to fully establish the efficacy and durability of COVID-19 vaccination in CLD and post-LT patients remain a major unmet need. As the pandemic continues to spread, unevenly, across the globe it remains unclear if cirrhosis or LT patients will require multiple vaccinations. Moreover, as newer variants of SARS-CoV-2 virus appear, particularly delta and omicron variants, further data about strain-specific effects and immunity are required to inform health policy. These questions are of urgent public health importance and will require addressing by hepatology community in the near future.

## Conclusions

**Fig. 4:**
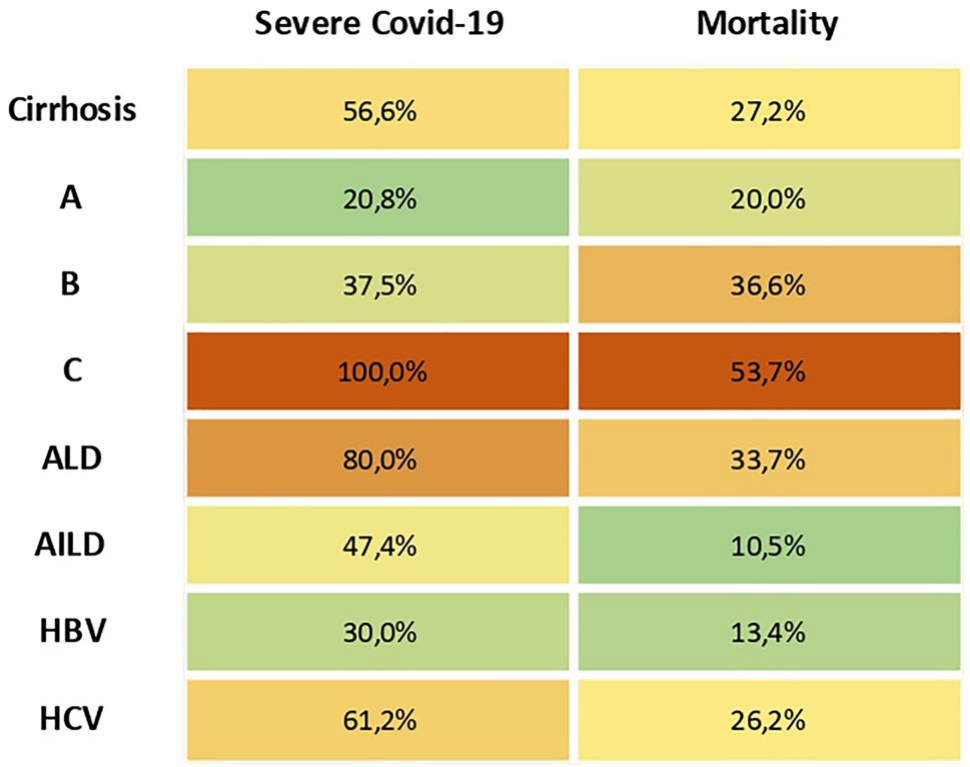
Risk of severe Covid-19 and overall mortality considering the weighted value of published studies, adjusted for the number of patients included. Risk of severe COVID-19 was a composite endpoint of need for hospitalization, ICU or ventilation for each aetiology and risk of liver injury in Child–Pugh class

Liver involvement is a common feature of SARS-CoV-2 infection that can affect COVID-19 prognosis. Although most current data are accrued from retrospective studies, it appears that patients with cirrhosis, ARLD or NALFD are at significantly increased risk of severe disease while the impact on patients with AILD or HBV infections is less clear (Fig. 4). Although the COVID-19 vaccination programme has re-opened much of society, emerging data suggest decreased efficacy of COVID-19 vaccines in patients with cirrhosis and more robust data are needed. Further public health recommendations or research strategies addressing COVID-19 in liver disease may be formulated based on the fundamental epidemiological data provided in this review.

## References

[1] Sanyaolu A, Okorie C, Marinkovic A (2020). Comorbidity and its impact on patients with COVID-19. SN Compr Clin Med.

[2] Marjot T, Webb GJ, Barritt AS (2021). COVID-19 and liver disease: mechanistic and clinical perspectives. Nat Rev Gastroenterol Hepatol.

[3] Zhao B, Ni C, Gao R (2020). Recapitulation of SARS-CoV-2 infection and cholangiocyte damage with human liver ductal organoids. Protein Cell.

[4] Yang L, Han Y, Nilsson-Payant BE (2020). A human pluripotent stem cell-based platform to study SARS-CoV-2 tropism and model virus infection in human cells and organoids. Cell Stem Cell.

[5] Faruqui S, Okoli FC, Olsen SK (2021). Cholangiopathy after severe COVID-19: clinical features and prognostic implications. Am J Gastroenterol.

[6] Chu H, Chan JF, Yuen TT (2020). Comparative tropism, replication kinetics, and cell damage profiling of SARS-CoV-2 and SARS-CoV with implications for clinical manifestations, transmissibility, and laboratory studies of COVID-19: an observational study. Lancet Microbe.

[7] Wang Y, Liu S, Liu H (2020). SARS-CoV-2 infection of the liver directly contributes to hepatic impairment in patients with COVID-19. J Hepatol.

[8] Bangash MN, Patel JM, Parekh D (2020). SARS-CoV-2: is the liver merely a bystander to severe disease?. J Hepatol.

[9] Meijnikman AS, Bruin S, Groen AK (2021). Increased expression of key SARS-CoV-2 entry points in multiple tissues in individuals with NAFLD. J Hepatol.

[10] Paizis G, Tikellis C, Cooper ME (2005). Chronic liver injury in rats and humans upregulates the novel enzyme angiotensin converting enzyme 2. Gut.

[11] Warner FJ, Rajapaksha H, Shackel N (2020). ACE2: from protection of liver disease to propagation of COVID-19. Clin Sci (Lond).

[12] McCarron S, Bathon B, Conlon DM (2021). Functional characterization of organoids derived from irreversibly damaged liver of patients with NASH. Hepatology.

[13] Clark R, Waters B, Stanfill AG (2021). Elevated liver function tests in COVID-19: causes, clinical evidence, and potential treatments. Nurse Pract.

[14] Da BL, Kushner T, El Halabi M (2020). Liver injury in hospitalized patients with COVID-19 correlates with hyper inflammatory response and elevated IL-6. Hepatol Commun.

[15] McConnell MJ, Kawaguchi N, Kondo R (2021). Liver injury in COVID-19 and IL-6 trans-signaling-induced endotheliopathy. J Hepatol.

[16] Bzeizi K, Abdulla M, Mohammed N, et al. Effect of COVID-19 on liver abnormalities: a systematic review and meta-analysis. Sci Rep. 10.1038/s41598-021-89513-9 PMC8134580 34012016

[17] Papic N, Pangercic A, Vargovic M (2012). Liver involvement during influenza infection: perspective on the 2009 influenza pandemic. Influenza Other Respir Viruses.

[18] Shafran N, Issachar A, Shochat T (2021). Abnormal liver tests in patients with SARS-CoV-2 or influenza—prognostic similarities and temporal disparities. JHEP Rep.

[19] Duchini A, Viernes ME, Nyberg LM (2000). Hepatic decompensation in patients with cirrhosis during infection with influenza A. Arch Intern Med.

[20] Bal CK, Bhatia V, Kumar S (2014). Influenza A/H1/N1/09 infection in patients with cirrhosis has a poor outcome: a case series. Indian J Gastroenterol.

[21] Marjot T, Moon AM, Cook JA (2021). Outcomes following SARS-CoV-2 infection in patients with chronic liver disease: an international registry study. J Hepatol.

[22] Singh S, Khan A (2020). Clinical characteristics and outcomes of coronavirus disease 2019 among patients with preexisting liver disease in the united states: a multicenter research network study. Gastroenterology.

[23] Ioannou GN, Liang PS, Locke E (2021). Cirrhosis and severe acute respiratory syndrome coronavirus 2 infection in US veterans: risk of infection, hospitalization, ventilation, and mortality. Hepatology.

[24] Mohammed A, Paranji N, Chen PH (2021). COVID-19 in chronic liver disease and liver transplantation: a clinical review. J Clin Gastroenterol.

[25] Sarin SK, Choudhury A, Lau GK (2020). Pre-existing liver disease is associated with poor outcome in patients with SARS CoV2 infection; The APCOLIS Study (APASL COVID-19 Liver Injury Spectrum Study). Hepatol Int.

[26] Macdonald S, Andreola F, Bachtiger P (2018). Cell death markers in patients with cirrhosis and acute decompensation. Hepatology.

[27] Vora SM, Lieberman J, Wu H (2021). Inflammasome activation at the crux of severe COVID-19. Nat Rev Immunol.

[28] Szabo G, Csak T (2012). Inflammasomes in liver diseases. J Hepatol.

[29] Soffientini U, Beaton N, Baweja S (2021). The lipopolysaccharide-sensing caspase(s)-4/11 are activated in cirrhosis and are causally associated with progression to multi-organ injury. Front Cell Dev Biol.

[30] Pan P, Shen M, Yu Z (2021). Author Correction: SARS-CoV-2 N protein promotes NLRP3 inflammasome activation to induce hyperinflammation. Nat Commun..

[31] Junqueira C, Crespo Ã, Ranjbar S, et al. SARS-CoV-2 infects blood monocytes to activate NLRP3 and AIM2 inflammasomes, pyroptosis and cytokine release. Res Sq [Preprint] 2021;rs.3.rs-153628

[32] Kim D, Adeniji N, Latt N (2021). Predictors of outcomes of COVID-19 in patients with chronic liver disease: US multi-center study. Clin Gastroenterol Hepatol.

[33] Szabo G, Saha B (2015). Alcohol's effect on host defense. Alcohol Res.

[34] Yang Z, Liu J, Zhou Y (2020). The effect of corticosteroid treatment on patients with coronavirus infection: a systematic review and meta-analysis. J Infect.

[35] Di Giorgio A, Nicastro E, Speziani C (2020). Health status of patients with autoimmune liver disease during SARS-CoV-2 outbreak in northern Italy. J Hepatol.

[36] Verhelst X, Somers N, Geerts A (2021). Health status of patients with autoimmune hepatitis is not affected by the SARS-CoV-2 outbreak in Flanders, Belgium. J Hepatol.

[37] Efe C, Dhanasekaran R, Lammert C (2021). Outcome of COVID-19 in patients with autoimmune hepatitis: an international multicenter study. Hepatology.

[38] Ji D, Qin E, Xu J (2020). Non-alcoholic fatty liver diseases in patients with COVID-19: a retrospective study. J Hepatol.

[39] Zheng KI, Gao F, Wang XB (2020). Letter to the EditOR Obesity as a risk factor for greater severity of COVID-19 in patients with metabolic associated fatty liver disease. Metabolism.

[40] Targher G, Mantovani A, Byrne CD (2020). Risk of severe illness from COVID-19 in patients with metabolic dysfunction-associated fatty liver disease and increased fibrosis scores. Gut.

[41] Liu J, Wang T, Cai Q (2020). Longitudinal changes of liver function and hepatitis B reactivation in COVID-19 patients with pre-existing chronic hepatitis B virus infection. Hepatol Res.

[42] Chen X, Jiang Q, Ma Z (2020). Clinical characteristics of hospitalized patients with SARS-CoV-2 and hepatitis B virus co-infection. Virol Sin.

[43] Rodríguez-Tajes S, Miralpeix A, Costa J (2021). Low risk of hepatitis B reactivation in patients with severe COVID-19 who receive immunosuppressive therapy. J Viral Hepat.

[44] Choe JW, Jung YK, Yim HJ (2022). Clinical effect of hepatitis B virus on COVID-19 infected patients: a nationwide population-based study using the health insurance review and assessment service database. J Korean Med Sci.

[45] Kang SH, Cho DH, Choi J (2021). Association between chronic hepatitis B infection and COVID-19 outcomes: a Korean nationwide cohort study. PLoS One.

[46] Ronderos D, Omar AMS, Abbas H (2021). Chronic hepatitis-C infection in COVID-19 patients is associated with in-hospital mortality. World J Clin Cases.

[47] Cerbu B, Pantea S, Bratosin F (2021). Liver impairment and hematological changes in patients with chronic hepatitis C and COVID-19: a retrospective study after one year of pandemic. Med (Kaunas).

[48] Butt AA, Yan P, Chotani RA (2021). Mortality is not increased in SARS-CoV-2 infected persons with hepatitis C virus infection. Liver Int.

[49] Lens S, Miquel M, Mateos-Muñoz B (2021). Epidemiological pattern, incidence, and outcomes of COVID-19 in liver transplant patients. J Hepatol.

[50] Colmenero J, Rodríguez-Perálvarez M, Salcedo M (2021). Epidemiological pattern, incidence, and outcomes of COVID-19 in liver transplant patients. J Hepatol.

[51] Webb GJ, Marjot T, Cook JA (2020). Outcomes following SARS-CoV-2 infection in liver transplant recipients: an international registry study. Lancet Gastroenterol Hepatol.

[52] Bhoori S, Rossi RE, Citterio D (2020). COVID-19 in long-term liver transplant patients: preliminary experience from an Italian transplant centre in Lombardy. Lancet Gastroenterol Hepatol.

[53] Webb GJ, Moon AM, Barnes E (2021). Age and comorbidity are central to the risk of death from COVID-19 in liver transplant recipients. J Hepatol.

[54] Rabiee A, Sadowski B, Adeniji N (2020). Liver injury in liver transplant recipients with coronavirus disease 2019 (COVID-19): US multicenter experience. Hepatology.

[55] John BV, Deng Y, Scheinberg A (2021). Association of BNT162b2 mRNA and mRNA-1273 vaccines With COVID-19 infection and hospitalization among patients with cirrhosis. JAMA Intern Med.

[56] Moon AM, Webb GJ, García-Juárez I (2021). SARS-CoV-2 infections among patients with liver disease and liver transplantation who received COVID-19 vaccination. Hepatol Commun.

[57] Wang J, Hou Z, Liu J (2021). Safety and immunogenicity of COVID-19 vaccination in patients with non-alcoholic fatty liver disease (CHESS2101): a multicenter study. J Hepatol.

[58] Thuluvath PJ, Robarts P, Chauhan M (2021). Analysis of antibody responses after COVID-19 vaccination in liver transplant recipients and those with chronic liver diseases. J Hepatol.

[59] Iavarone M, Tosetti G, Facchetti F (2021). Delayed and suboptimal response to two doses of SARS-CoV-2 messenger RNA vaccine in European patients with compensated and decompensated cirrhosis of difference aetiologies. Hepatology.

[60] Rabinowich L, Grupper A, Baruch R (2021). Low immunogenicity to SARS-CoV-2 vaccination among liver transplant recipients. J Hepatol.

